# Supplementary material for: Arginine-Enhanced Termitomyces Mycelia: Improvement in Growth and Lignocellulose Degradation Capabilities
Source: Foods. 2025 Jan 23;14(3):361. doi: 10.3390/foods14030361 (PMC11817607; doi:10.3390/foods14030361)
Supplement: Supplementary file 1 [file foods-14-00361-s001.zip › foods-3352056-supplementary.pdf]

Supplementary Figures&Table

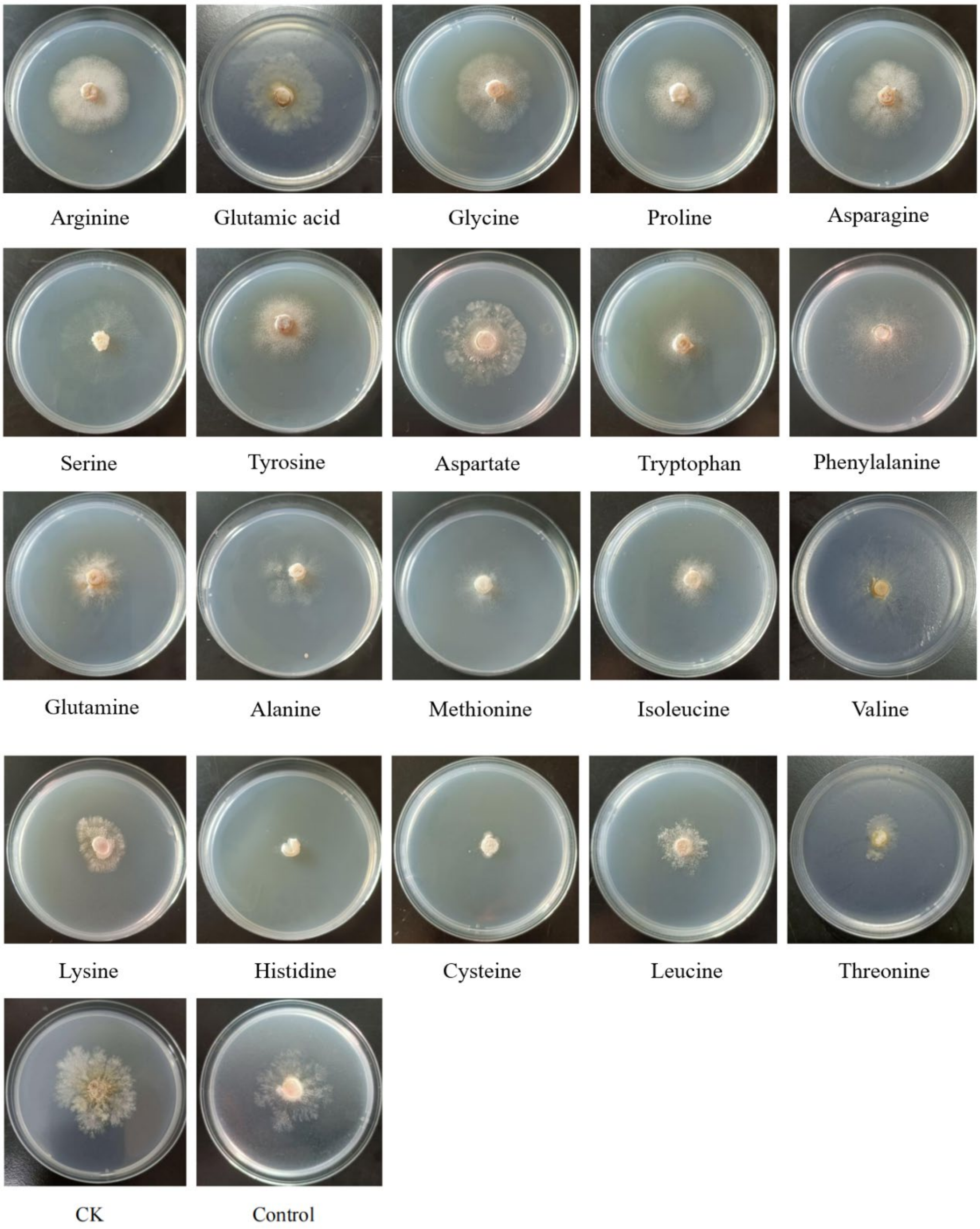

Figure S1 Morphology of plate colonies of *Termitomyces* under different amino acid cultures

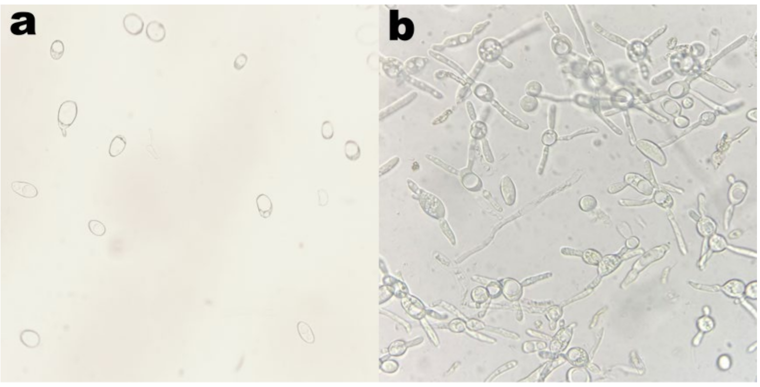

Figure S2 Micromorphological observation of spore germination

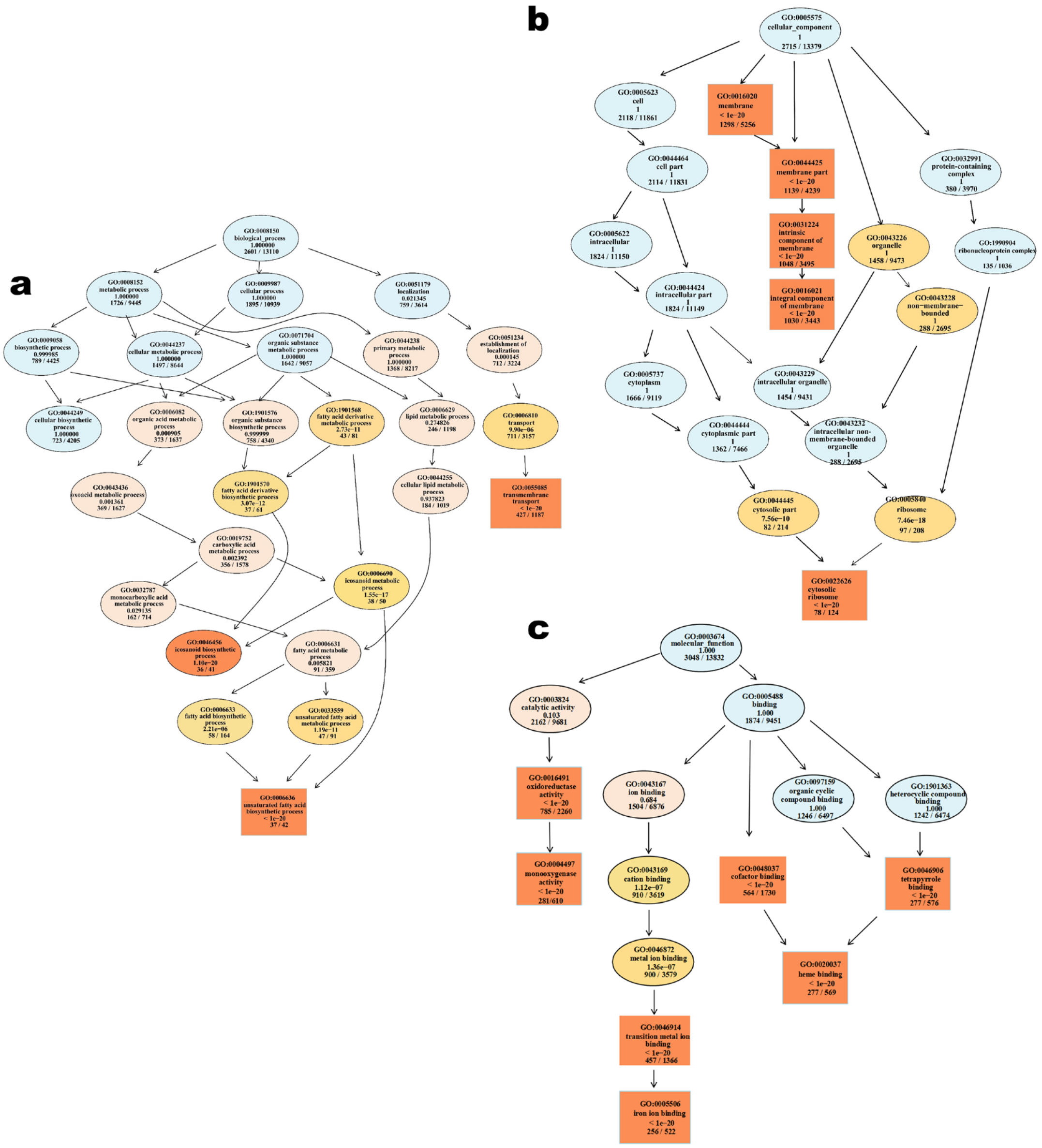

Figure S3 Thumbnails view of DAG between CK vs. Arg. (a) DAG on biological process. (b) DAG on cellular component. (c) DAG on molecular function. Note: GO term is a node; the parentage is an arrow. The degree of enrichment is higher along with the darker of color.

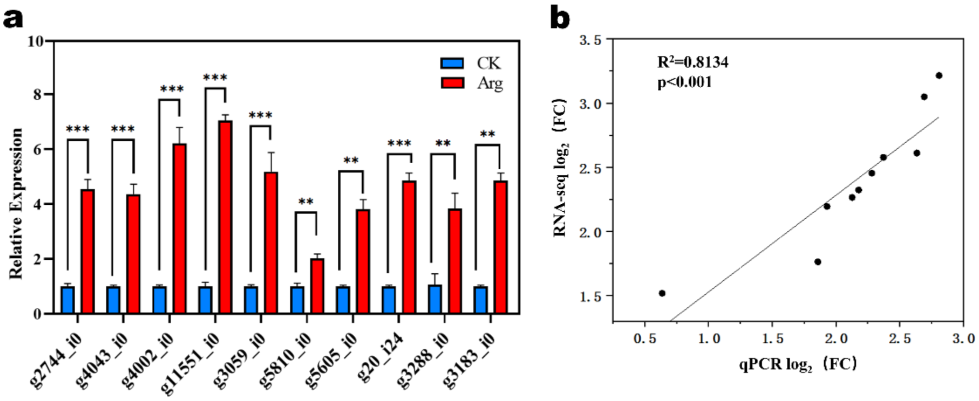

Figure S4 (a)Expression of key genes related to the metabolic pathway (qPCR validation); values are expressed as mean ± SEM (n = 3), *p < 0.05 and **p < 0.01;(b) The correlation between results from qPCR and RNA-seq.

Table S1 The CAZymes family in the developmental transcriptome

CAYz	count of family	count of gene	major family
GHs	80	648	GH16、GH79、GH18、GH3、GH92、GH2、GH115、GH47、GH5_9、GH31、GH10、GH71、GH35、GH5_50、GH7、GH27、GH28、GH78、GH17、GH29、GH5_5、GH6、GH76、GH88、GH15、GH5_12、GH63、GH81、GH95、GH105、GH13_1、GH13_22、GH74、GH85、GH30_3、GH37、GH5_30、GH51、GH53、GH109、GH25、GH30_2、GH99、GH1、GH11、GH43_30、GH5_7、GH125、GH13_25、GH13_5、GH13_8、GH133、GH33、GH43_13、GH5_46、GH12、GH128、GH13_19、GH13_30、GH131、GH142、GH143、GH44、GH5、GH5_24、GH5_35、GH5_36、GH57、GH72、GH116、GH120、GH124、GH127、GH13_7、GH23、GH43_24、GH5_13、GH5_22、GH9、GH96

GTs	36	416	GT41、GT2、GT90、GT1、GT39、GT68、GT15、GT4、GT32、GT48、GT58、GT66、GT33、GT57、GT22、GT26、GT3、GT31、GT50、GT8、GT20、GT35、GT65、GT2_Cellulose_synt、GT21、GT23、GT28、GT5、GT83、GT24、GT25、GT30、GT38、GT49、GT80、GT87
AAs	17	313	AA1、AA3_2、AA1_1、AA3、AA3_1、AA7、AA5_1、AA9、AA3_3、AA2、AA8、AA5、AA1_2、AA4、AA3_4、AA6、AA5_2
CBMs	16	75	CBM67、CBM13、CBM21、CBM50、CBM19、CBM1、CBM16、CBM57、CBM12、CBM14、CBM22、CBM32、CBM44、CBM5、CBM53、CBM60
CEs	10	70	CE10、CE1、CE9、CE7、CE4、CE16、CE5、CE12、CE8、CE15
PLs	17	64	PL8、PL14、PL25、PL1、PL11、PL14_4、PL14_5、PL3_2、PL5_1、PL1_11、PL1_7、PL10、PL12、PL27、PL17_1、PL4_1、PL5

Table S2 The genes and primers required used for qRT-PCR validation.

Genes ID	Description	Forward sequence (5'-3')	Reverse sequence (5'-3')
g4002_i0	cellulase,endoglucanase	ACTATCAAGTCAACCGAGGC	GAGACTACATCAAGGCAACG
g3183_i0	GH5_5	TCAATGGAAGAAGGCGAAGG	CGAGTCTGGTGCCGAGTTTG
g3059_i0	beta-glucosidase	CTCATTATTTGCTGTGGGTC	CTCATTATTTGCTGTGGGTC
g20_i24	GH3	AGCAGAGCCTCCGCCAGAAA	AGCAGAGCCTCCGCCAGAAA
g11551_i0	cellulose 1,4-beta-cellobiosidase	AAGAAGGCGTTTGGAGATAG	GGGTCAGCATTGATAGGATAA
g2744_i0	pyruvate dehydrogenase	TTCCACTTGCCCTTGGTTGC	TTGTTGGTGCTCATGTTCCC

g3288_i0	pyruvate decarboxylase	GATGAAACGCTCAATAGTGTAG	TGGAGATGGAAGCCTGTAAG
g4043_i0	aldehyde dehydrogenase (NAD+)	CAAACGCCCAATCATAAGTC	TGCTAAACAACCTCGCAAAC
g5605_i0	alcohol dehydrogenase	CGCGTCTTGTCGGTTTCCTA	CACCACTGCTTCCTCCTCTG
g5810_i0	arginase	ATGTTCTCCCACTGCTTTCG	ACCCTCCTGTTGGCATTCTG

Table S3. The characteristic absorption peak of infrared spectrum of Corn straw and its attribution.

Absorption peak/cm^{-1}	Attribution
835	Bending vibrations outside the C-H plane of the S ring and H ring (lignin syringyl vibration)
1040	Stretching vibration of carbohydrate (cellulose, hemicellulose) or polysaccharide C-O
1215	Stretching vibration of phenolic hydroxyl C-O on the benzene ring
1240	The ester bond between hemicellulose and lignin
1325	Stretching vibration of C-O on syringyl aromatic ring
1372	Bending vibrations of C-H on cellulose and hemicellulose
1429	Stretching vibration of lignin, aliphatic compoun and C-N
1515	Stretching vibration of C=C-C on the benzene ring of lignin
1613	Vibration of lignin aromatic skeletal
1641	Stretching vibration of C=O associated with aromatic rings in lignin
2352	N≡N stretching vibration
2925	Contraction of C-H in cellulose
3333	Hydrogen bond association, which includes the stretching vibration of the hydrogen bond formed by -OH and N-H in amino acids, as well as cellulose, hemicellulose, starch and monosaccharide, et al
